# 1-(*tert*-But­oxy­carbon­yl)piperidine-4-carb­oxy­lic acid

**DOI:** 10.1107/S1600536811030145

**Published:** 2011-08-02

**Authors:** Hoong-Kun Fun, Suhana Arshad, Dinesh, S. Vivek, G. K. Nagaraja

**Affiliations:** a X-ray Crystallography Unit, School of Physics, Universiti Sains Malaysia, 11800 USM, Penang, Malaysia; b Department of Chemistry, Mangalore University, Karnataka, India

## Abstract

In the title compound, C_11_H_19_NO_4_, the piperidine ring adopts a chair conformation. In the crystal, mol­ecules are linked by inter­molecular O—H⋯O and C—H⋯O hydrogen bonds, forming a layer parallel to the *bc* plane.

## Related literature

For general background and application of helical peptides, see: Albrecht & Stortz (2005 ▶); Garner & Harding (2007 ▶); Wang *et al.* (2008 ▶); Walensky *et al.* (2004 ▶); Boal *et al.* (2007 ▶). For bond-length data, see: Allen *et al.* (1987 ▶). For ring conformations, see: Cremer & Pople (1975 ▶). For stability of the temperature controller used for data collection, see: Cosier & Glazer (1986 ▶).
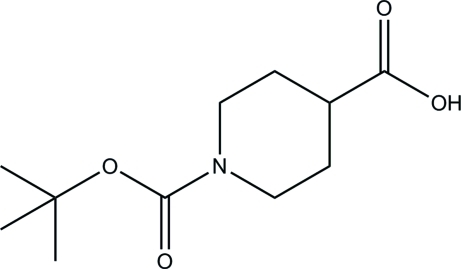


## Experimental

### 

#### Crystal data

C_11_H_19_NO_4_
*M* *_r_* = 229.27
Monoclinic, 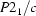

*a* = 10.7006 (3) Å
*b* = 6.5567 (2) Å
*c* = 17.9297 (6) Å
β = 104.564 (2)°
*V* = 1217.54 (6) Å^3^
*Z* = 4
Mo *K*α radiation
μ = 0.10 mm^−1^
*T* = 100 K
0.57 × 0.21 × 0.08 mm

#### Data collection

Bruker SMART APEXII CCD area-detector diffractometer
Absorption correction: multi-scan (*SADABS*; Bruker, 2009 ▶) *T*_min_ = 0.948, *T*_max_ = 0.993
5360 measured reflections
2116 independent reflections
1762 reflections with *I* > 2σ(*I*)
*R*_int_ = 0.027

#### Refinement

*R*[*F* ^2^ > 2σ(*F* ^2^)] = 0.039
*wR*(*F* ^2^) = 0.092
*S* = 1.07
2116 reflections
152 parameters
H atoms treated by a mixture of independent and constrained refinement
Δρ_max_ = 0.18 e Å^−3^
Δρ_min_ = −0.22 e Å^−3^

### 

Data collection: *APEX2* (Bruker, 2009 ▶); cell refinement: *SAINT* (Bruker, 2009 ▶); data reduction: *SAINT*; program(s) used to solve structure: *SHELXTL* (Sheldrick, 2008 ▶); program(s) used to refine structure: *SHELXTL*; molecular graphics: *SHELXTL*; software used to prepare material for publication: *SHELXTL* and *PLATON* (Spek, 2009 ▶).

## Supplementary Material

Crystal structure: contains datablock(s) global, I. DOI: 10.1107/S1600536811030145/is2754sup1.cif

Structure factors: contains datablock(s) I. DOI: 10.1107/S1600536811030145/is2754Isup2.hkl

Supplementary material file. DOI: 10.1107/S1600536811030145/is2754Isup3.cml

Additional supplementary materials:  crystallographic information; 3D view; checkCIF report

## Figures and Tables

**Table 1:** Hydrogen-bond geometry (Å, °)

*D*—H⋯*A*	*D*—H	H⋯*A*	*D*⋯*A*	*D*—H⋯*A*
O4—H1O4⋯O1^i^	0.86 (2)	1.82 (2)	2.6562 (16)	164 (2)
C5—H5*B*⋯O4^ii^	0.99	2.56	3.476 (2)	154

Symmetry codes: (i) 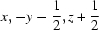; (ii) 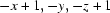.

**Fig. 1.**
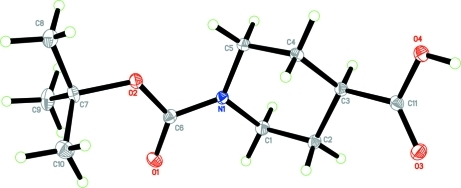
The molecular structure of the title compound, showing 30% probability displacement ellipsoids and the atom-numbering scheme.

**Fig. 2.**
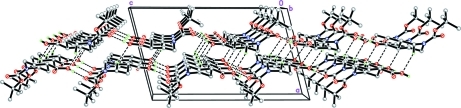
The crystal packing of the title compound showing a two-molecular thick sheet. Dashed lines represent the intermolecular hydrogen bonds.

## supplementary crystallographic information

### Comment

An intramolecular side-chain metal ligation is a useful method for stabilizing β-sheet, turn and helical structures in short peptides (Albrecht & Stortz, 2005; Garner & Harding, 2007). The stabilization of helical structure may enhance biological activities and protease resistance *in vitro* or *in vivo* (Wang *et al.*, 2008; Walensky *et al.*, 2004). The present compound, 1-(*tert*-butoxycarbonyl)piperidine-4-carboxylic acid, may be used as a rigid backbone to design the metal-ligating 3_10_-helical peptide. In this way we may be able to design, synthesize and characterize a dynamically optically inactive 3_10_-helical peptide which possess a metal-chelating ability (Boal *et al.*, 2007).

In the molecular structure (Fig. 1), the piperidine ring (N1/C1–C5) adopts a chair conformation with puckering amplitude Q = 0.5505 (16) Å, Θ= 179.17 (18)° and φ= 90 (11)° (Cremer & Pople, 1975). The bond lengths (Allen *et al.*, 1987) and angles are within normal ranges.

The crystal packing is shown in Fig. 2. The molecules are linked by intermolecular O4—H1O4···O1 and C5—H5B···O4 (Table 1) hydrogen bonds, forming two-molecular sheets parallel to the *bc* plane.

### Experimental

To isopicotinic acid (1 eq) dissolved in a dichloromethane (10 ml), triethylamine (3 eq) was added. The reaction mixture was stirred at room temperature for half an hour. BOC-anhydride (2 eq) was then added and the reaction mixture heated at 40 °C for 12 h. Completion of the reaction was confirmed by TLC and the solvent content was evaporated away under reduced pressure. The reaction mixture was acidified with diluted. HCl and the solid obtained was filtered off. *M. p.*: 135–137 °C.

### Refinement

H1O4 atom attached to the O atom was located in a difference map and refined freely [O—H = 0.86 (2) Å]. The remaining H atoms were positioned geometrically (C—H = 0.98–1.00 Å) and refined using a riding model with *U*_iso_(H) = 1.2 or 1.5*U*_eq_(C). A rotating group model was applied to the methyl groups.

### Crystal data

**Table:**

C_11_H_19_NO_4_	*F*(000) = 496
*M**_r_* = 229.27	*D*_x_ = 1.251 Mg m^−^^3^
Monoclinic, *P*2_1_/*c*	Mo *K*α radiation, λ = 0.71073 Å
Hall symbol: -P 2ybc	Cell parameters from 1750 reflections
*a* = 10.7006 (3) Å	θ = 2.4–28.5°
*b* = 6.5567 (2) Å	µ = 0.10 mm^−^^1^
*c* = 17.9297 (6) Å	*T* = 100 K
β = 104.564 (2)°	Plate, colourless
*V* = 1217.54 (6) Å^3^	0.57 × 0.21 × 0.08 mm
*Z* = 4	

### Data collection

**Table:**

Bruker SMART APEXII CCD area-detector diffractometer	2116 independent reflections
Radiation source: fine-focus sealed tube	1762 reflections with *I* > 2σ(*I*)
graphite	*R*_int_ = 0.027
φ and ω scans	θ_max_ = 25.0°, θ_min_ = 2.0°
Absorption correction: multi-scan (*SADABS*; Bruker, 2009)	*h* = −12→11
*T*_min_ = 0.948, *T*_max_ = 0.993	*k* = −7→7
5360 measured reflections	*l* = −16→21

### Refinement

**Table:**

Refinement on *F*^2^	Primary atom site location: structure-invariant direct methods
Least-squares matrix: full	Secondary atom site location: difference Fourier map
*R*[*F*^2^ > 2σ(*F*^2^)] = 0.039	Hydrogen site location: inferred from neighbouring sites
*wR*(*F*^2^) = 0.092	H atoms treated by a mixture of independent and constrained refinement
*S* = 1.07	*w* = 1/[σ^2^(*F*_o_^2^) + (0.0362*P*)^2^ + 0.3737*P*] where *P* = (*F*_o_^2^ + 2*F*_c_^2^)/3
2116 reflections	(Δ/σ)_max_ < 0.001
152 parameters	Δρ_max_ = 0.18 e Å^−^^3^
0 restraints	Δρ_min_ = −0.22 e Å^−^^3^

### Special details

**Table:**

Experimental. The crystal was placed in the cold stream of an Oxford Cryosystems Cobra open-flow nitrogen cryostat (Cosier & Glazer, 1986) operating at 100.0 (1) K.
Geometry. All e.s.d.'s (except the e.s.d. in the dihedral angle between two l.s. planes) are estimated using the full covariance matrix. The cell e.s.d.'s are taken into account individually in the estimation of e.s.d.'s in distances, angles and torsion angles; correlations between e.s.d.'s in cell parameters are only used when they are defined by crystal symmetry. An approximate (isotropic) treatment of cell e.s.d.'s is used for estimating e.s.d.'s involving l.s. planes.
Refinement. Refinement of *F*^2^ against ALL reflections. The weighted *R*-factor *wR* and goodness of fit *S* are based on *F*^2^, conventional *R*-factors *R* are based on *F*, with *F* set to zero for negative *F*^2^. The threshold expression of *F*^2^ > σ(*F*^2^) is used only for calculating *R*-factors(gt) *etc*. and is not relevant to the choice of reflections for refinement. *R*-factors based on *F*^2^ are statistically about twice as large as those based on *F*, and *R*- factors based on ALL data will be even larger.

### Fractional atomic coordinates and isotropic or equivalent isotropic displacement parameters (Å^2^)

**Table:**

	*x*	*y*	*z*	*U*_iso_*/*U*_eq_	
O1	0.26358 (11)	−0.05214 (18)	0.13924 (6)	0.0249 (3)	
O2	0.20095 (11)	0.21800 (16)	0.20083 (6)	0.0229 (3)	
O3	0.34156 (12)	−0.53547 (17)	0.46087 (7)	0.0265 (3)	
O4	0.35972 (11)	−0.25776 (18)	0.53522 (6)	0.0244 (3)	
N1	0.34480 (13)	−0.0015 (2)	0.26790 (7)	0.0188 (3)	
C1	0.42896 (16)	−0.1805 (2)	0.27802 (9)	0.0213 (4)	
H1A	0.5202	−0.1364	0.2948	0.026*	
H1B	0.4167	−0.2525	0.2282	0.026*	
C2	0.39922 (16)	−0.3257 (2)	0.33783 (9)	0.0195 (4)	
H2A	0.4628	−0.4385	0.3473	0.023*	
H2B	0.3123	−0.3855	0.3176	0.023*	
C3	0.40397 (15)	−0.2158 (2)	0.41359 (9)	0.0178 (4)	
H3A	0.4949	−0.1705	0.4361	0.021*	
C4	0.31744 (15)	−0.0254 (2)	0.39936 (9)	0.0186 (4)	
H4A	0.2259	−0.0677	0.3819	0.022*	
H4B	0.3284	0.0509	0.4482	0.022*	
C5	0.35063 (16)	0.1126 (2)	0.33908 (9)	0.0202 (4)	
H5A	0.2891	0.2280	0.3280	0.024*	
H5B	0.4385	0.1690	0.3591	0.024*	
C6	0.26959 (15)	0.0473 (2)	0.19822 (9)	0.0190 (4)	
C7	0.10863 (15)	0.2991 (3)	0.13127 (9)	0.0221 (4)	
C8	0.06014 (17)	0.4903 (3)	0.16286 (10)	0.0288 (4)	
H8A	0.0189	0.4528	0.2039	0.043*	
H8B	−0.0027	0.5596	0.1214	0.043*	
H8C	0.1330	0.5818	0.1837	0.043*	
C9	0.17864 (18)	0.3540 (3)	0.07020 (10)	0.0330 (5)	
H9A	0.2538	0.4391	0.0932	0.049*	
H9B	0.1201	0.4294	0.0285	0.049*	
H9C	0.2073	0.2291	0.0495	0.049*	
C10	0.00063 (17)	0.1463 (3)	0.10281 (10)	0.0303 (4)	
H10A	−0.0400	0.1137	0.1446	0.046*	
H10B	0.0361	0.0215	0.0861	0.046*	
H10C	−0.0639	0.2048	0.0594	0.046*	
C11	0.36557 (14)	−0.3565 (2)	0.47085 (9)	0.0183 (4)	
H1O4	0.333 (2)	−0.340 (4)	0.5652 (13)	0.058 (7)*	

### Atomic displacement parameters (Å^2^)

**Table:**

	*U*^11^	*U*^22^	*U*^33^	*U*^12^	*U*^13^	*U*^23^
O1	0.0329 (7)	0.0282 (7)	0.0148 (6)	0.0004 (5)	0.0084 (5)	−0.0034 (5)
O2	0.0274 (6)	0.0221 (6)	0.0170 (6)	0.0056 (5)	0.0018 (5)	0.0005 (5)
O3	0.0390 (7)	0.0190 (7)	0.0240 (7)	−0.0008 (5)	0.0125 (6)	0.0008 (5)
O4	0.0353 (7)	0.0226 (7)	0.0175 (6)	−0.0008 (5)	0.0109 (6)	0.0003 (5)
N1	0.0264 (8)	0.0164 (7)	0.0138 (7)	0.0028 (6)	0.0056 (6)	0.0008 (6)
C1	0.0253 (9)	0.0206 (9)	0.0201 (9)	0.0064 (7)	0.0099 (7)	−0.0003 (7)
C2	0.0234 (9)	0.0174 (8)	0.0191 (9)	0.0044 (7)	0.0082 (7)	0.0014 (7)
C3	0.0181 (8)	0.0183 (9)	0.0168 (8)	−0.0005 (7)	0.0043 (7)	0.0012 (7)
C4	0.0218 (9)	0.0189 (9)	0.0153 (8)	0.0010 (7)	0.0051 (7)	−0.0019 (7)
C5	0.0271 (9)	0.0166 (8)	0.0164 (8)	0.0002 (7)	0.0048 (7)	−0.0011 (7)
C6	0.0215 (9)	0.0184 (9)	0.0190 (9)	−0.0017 (7)	0.0086 (7)	0.0033 (7)
C7	0.0205 (9)	0.0276 (9)	0.0159 (8)	0.0012 (7)	0.0006 (7)	0.0046 (7)
C8	0.0264 (10)	0.0274 (10)	0.0296 (10)	0.0053 (8)	0.0017 (8)	0.0026 (8)
C9	0.0311 (10)	0.0395 (11)	0.0297 (10)	0.0074 (9)	0.0101 (8)	0.0152 (9)
C10	0.0257 (10)	0.0341 (11)	0.0295 (10)	−0.0009 (8)	0.0039 (8)	−0.0021 (8)
C11	0.0167 (8)	0.0204 (9)	0.0170 (8)	0.0034 (7)	0.0025 (7)	−0.0003 (7)

### Geometric parameters (Å, °)

**Table:**

O1—C6	1.2303 (18)	C4—C5	1.519 (2)
O2—C6	1.3457 (19)	C4—H4A	0.9900
O2—C7	1.4815 (18)	C4—H4B	0.9900
O3—C11	1.2050 (19)	C5—H5A	0.9900
O4—C11	1.3384 (18)	C5—H5B	0.9900
O4—H1O4	0.86 (2)	C7—C9	1.517 (2)
N1—C6	1.344 (2)	C7—C10	1.518 (2)
N1—C1	1.463 (2)	C7—C8	1.519 (2)
N1—C5	1.4669 (19)	C8—H8A	0.9800
C1—C2	1.526 (2)	C8—H8B	0.9800
C1—H1A	0.9900	C8—H8C	0.9800
C1—H1B	0.9900	C9—H9A	0.9800
C2—C3	1.527 (2)	C9—H9B	0.9800
C2—H2A	0.9900	C9—H9C	0.9800
C2—H2B	0.9900	C10—H10A	0.9800
C3—C11	1.512 (2)	C10—H10B	0.9800
C3—C4	1.537 (2)	C10—H10C	0.9800
C3—H3A	1.0000		
			
C6—O2—C7	121.57 (12)	C4—C5—H5B	109.6
C11—O4—H1O4	109.2 (15)	H5A—C5—H5B	108.1
C6—N1—C1	120.84 (13)	O1—C6—N1	124.22 (15)
C6—N1—C5	124.88 (13)	O1—C6—O2	124.04 (15)
C1—N1—C5	114.27 (12)	N1—C6—O2	111.74 (13)
N1—C1—C2	110.93 (12)	O2—C7—C9	110.31 (13)
N1—C1—H1A	109.5	O2—C7—C10	109.63 (13)
C2—C1—H1A	109.5	C9—C7—C10	112.73 (15)
N1—C1—H1B	109.5	O2—C7—C8	101.56 (12)
C2—C1—H1B	109.5	C9—C7—C8	110.50 (14)
H1A—C1—H1B	108.0	C10—C7—C8	111.56 (14)
C1—C2—C3	111.36 (13)	C7—C8—H8A	109.5
C1—C2—H2A	109.4	C7—C8—H8B	109.5
C3—C2—H2A	109.4	H8A—C8—H8B	109.5
C1—C2—H2B	109.4	C7—C8—H8C	109.5
C3—C2—H2B	109.4	H8A—C8—H8C	109.5
H2A—C2—H2B	108.0	H8B—C8—H8C	109.5
C11—C3—C2	111.22 (13)	C7—C9—H9A	109.5
C11—C3—C4	110.67 (12)	C7—C9—H9B	109.5
C2—C3—C4	110.60 (13)	H9A—C9—H9B	109.5
C11—C3—H3A	108.1	C7—C9—H9C	109.5
C2—C3—H3A	108.1	H9A—C9—H9C	109.5
C4—C3—H3A	108.1	H9B—C9—H9C	109.5
C5—C4—C3	111.29 (12)	C7—C10—H10A	109.5
C5—C4—H4A	109.4	C7—C10—H10B	109.5
C3—C4—H4A	109.4	H10A—C10—H10B	109.5
C5—C4—H4B	109.4	C7—C10—H10C	109.5
C3—C4—H4B	109.4	H10A—C10—H10C	109.5
H4A—C4—H4B	108.0	H10B—C10—H10C	109.5
N1—C5—C4	110.43 (12)	O3—C11—O4	122.99 (15)
N1—C5—H5A	109.6	O3—C11—C3	125.29 (14)
C4—C5—H5A	109.6	O4—C11—C3	111.72 (13)
N1—C5—H5B	109.6		
			
C6—N1—C1—C2	−123.01 (15)	C1—N1—C6—O2	−179.20 (13)
C5—N1—C1—C2	56.56 (17)	C5—N1—C6—O2	1.3 (2)
N1—C1—C2—C3	−53.55 (17)	C7—O2—C6—O1	1.2 (2)
C1—C2—C3—C11	176.11 (13)	C7—O2—C6—N1	−178.49 (13)
C1—C2—C3—C4	52.71 (17)	C6—O2—C7—C9	−61.40 (18)
C11—C3—C4—C5	−177.27 (13)	C6—O2—C7—C10	63.30 (17)
C2—C3—C4—C5	−53.56 (17)	C6—O2—C7—C8	−178.59 (13)
C6—N1—C5—C4	122.38 (16)	C2—C3—C11—O3	4.2 (2)
C1—N1—C5—C4	−57.18 (17)	C4—C3—C11—O3	127.55 (17)
C3—C4—C5—N1	54.71 (17)	C2—C3—C11—O4	−175.19 (13)
C1—N1—C6—O1	1.1 (2)	C4—C3—C11—O4	−51.83 (17)
C5—N1—C6—O1	−178.39 (14)		

### Hydrogen-bond geometry (Å, °)

**Table:**

*D*—H···*A*	*D*—H	H···*A*	*D*···*A*	*D*—H···*A*
O4—H1O4···O1^i^	0.86 (2)	1.82 (2)	2.6562 (16)	164 (2)
C5—H5B···O4^ii^	0.99	2.56	3.476 (2)	154.

Symmetry codes: (i) *x*, −*y*−1/2, *z*+1/2; (ii) −*x*+1, −*y*, −*z*+1.
